# Allosteric cross-talk between the hydrophobic cleft and the BH4 domain of Bcl-2 in control of inositol 1,4,5-trisphosphate receptor activity

**DOI:** 10.37349/etat.2022.00088

**Published:** 2022-06-28

**Authors:** George Shapovalov, Abigaël Ritaine, Nadege Charlene Essonghe, Ian de Ridder, Hristina Ivanova, Spyridoula Karamanou, Anastassios Economou, Geert Bultynck, Roman Skryma, Natalia Prevarskaya

**Affiliations:** 1 Univ. Lille, Inserm, U1003 - PHYCEL - Physiologie Cellulaire, F-59000 Lille, France; 2 Laboratory of Excellence, Ion Channels Science and Therapeutics, 59655 Villeneuve d’Ascq, France; 3 KU Leuven, Laboratory of Molecular and Cellular Signaling, Department of Cellular and Molecular Medicine, Campus Gasthuisberg O/N-I bus 802, Herestraat 49, B-3000 Leuven, Belgium; 4 KU Leuven, Department of Microbiology and Immunology, Rega Institute of Medical Research, Laboratory of Molecular Bacteriology, Herestraat 49, B-3000 Leuven, Belgium; Regina Elena National Cancer Institute, Italy

**Keywords:** Cancer, calcium, Bcl-2, IP_3_R, organelle membrane-derived patch-clamp, molecular dynamics

## Abstract

**Aim:**

Inositol 1,4,5-trisphosphate receptor (IP_3_R) is a ubiquitous calcium (Ca^2+^) channel involved in the regulation of cellular fate and motility. Its modulation by anti-apoptotic protein B-cell lymphoma 2 (Bcl-2) plays an important role in cancer progression. Disrupting this interaction could overcome apoptosis avoidance, one of the hallmarks of cancer, and is, thus, of great interest. Earlier reports have shown the involvement of both the Bcl-2 homology 4 (BH4) and the transmembrane domains (TMDs) of Bcl-2 in regulating IP_3_R activity, while the Bcl-2 hydrophobic cleft was associated primarily with its anti-apoptotic and IP_3_R-independent action at the mitochondria (Oncotarget. 2016;7:55704–20. doi: 10.18632/oncotarget.11005). The aim of this study was to investigate how targeting the BH3 hydrophobic cleft of Bcl-2 affects IP_3_R:Bcl-2 interaction.

**Methods:**

Organelle membrane-derived (OMD) patch-clamp and circular dichroism (CD) thermal melting experiments were used to elucidate the effects of the ABT-199 (venetoclax) on the IP_3_R:Bcl-2 interaction. Molecular dynamics (MD) simulations of free and ABT-199 bound Bcl-2 were used to propose a molecular model of such interaction.

**Results:**

It was shown that occlusion of Bcl-2’s hydrophobic cleft by the drug ABT-199 finely modulates IP_3_R gating in the low open probability (P_o_) regime, characteristic of the basal IP_3_R activity in non-excited cells. Complementary MD simulations allowed to propose a model of this modulation, involving an allosteric interaction with the BH4 domain on the opposite side of Bcl-2.

**Conclusions:**

Bcl-2 is an important regulator of IP_3_R activity and, thus of Ca^2+^ release from internal stores and associated processes, including cellular proliferation and death. The presence of multiple regulatory domains in both proteins suggests a complex interaction. Thus, it was found that the occlusion of the hydrophobic cleft of Bcl-2 by ABT-199 disrupts IP_3_R activity, leading to Bcl-2 rebinding with smaller affinity and lesser inhibitory effect. MDs simulations of free and ABT-199 bound Bcl-2 propose a molecular model of such disruption, involving an allosteric interaction with the BH4 domain on the opposite side of Bcl-2.

## Introduction

The inositol 1,4,5-trisphosphate receptor (IP_3_R) is an intracellular ligand-gated calcium (Ca^2+^) permeable channel that is located primarily at the endoplasmic reticulum (ER) membrane and is ubiquitously expressed. Its major role in intracellular Ca^2+^ dynamics determines its involvement in multiple cellular functions such as apoptosis, contraction, cell motility, proliferation, and migration [1–3]. As such, dysregulation of its activity can affect the initiation or progression of serious diseases, such as cancer [4, 5]. IP_3_R channels open upon binding of their physiological ligand inositol 1,4,5-trisphosphate (IP_3_). The activity of IP_3_R is further modulated by Ca^2+^, ATP, and H^+^, by its phosphorylation and other modifications, or by changes in its redox status [6–9]. Furthermore, the remarkable richness of the IP_3_R regulation is achieved via its interaction with partner proteins [10], of which many have functions in cell fate decisions [11]. These proteins can directly alter the IP_3_R-mediated Ca^2+^ flux by impacting IP_3_R gating or stability [12, 13].

**Figure 1.**
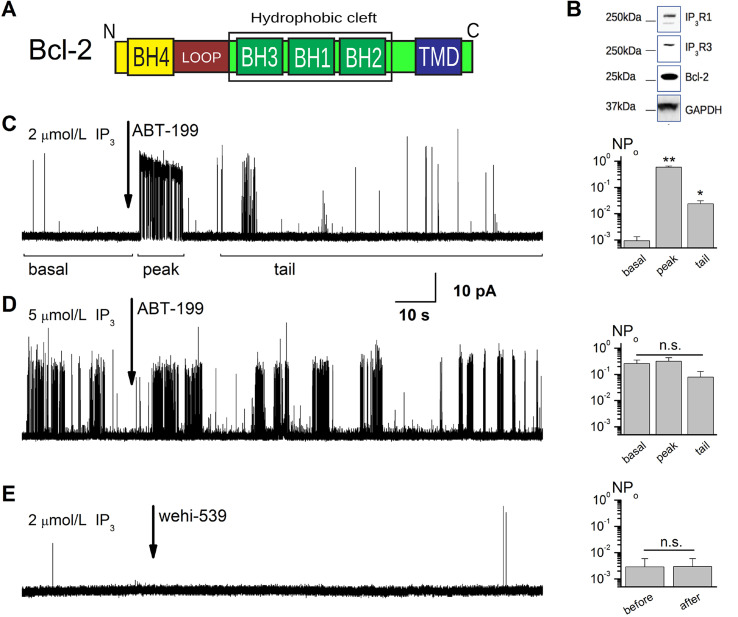
ABT-199 disturbs the Bcl-2-dependant inhibition of IP_3_R in conditions of low channel activity. (A). A schematic representation of the Bcl-2 subunit composition. Letters N and C mark the N- and C-terminal ends of the protein. (B). Expression levels of IP_3_R1, IP_3_R3, and Bcl-2 proteins are illustrated by western blot (WB) analysis of the ER fractions from the Bcl-2 overexpressing WEHI7.2 cells. (C, D). Sample traces showing the effect of application of 1 μmol/L ABT-199 to the patches exhibiting IP_3_R activity stimulated by 2 μmol/L (C) and 5 μmol/L (D) IP_3_. Notice the complex nature of the IP_3_R activity at characteristic open probability (P_o_) in panel C, with principal regions indicated by horizontal bars marking the regions of “basal”, “peak” (acute ABT-199 effect), and “tail” steady-state activity following ABT-199 application. Note also that such a complex response is masked by the elevated activity level of IP_3_R after stimulation by higher IP_3_ concentration ([IP_3_]) in panel D. (E). Sample traces showing the effect of application of 5 nmol/L wehi-539 to the patches exhibiting IP_3_R activity stimulated by 2 μmol/L IP_3_. Notice the absence of the complex IP_3_R response to the application of this Bcl-Xl specific antagonist. Barplots on the right of panels (C–E) summarize the average P_o_ for the presented conditions (*n* = 5, 6, and 7 correspondingly). *: *P* < 0.05; **: *P* ≤ 0.01; GADPH: glyceraldehyde-3-phosphate dehydrogenase; n.s.: no significant difference; pA: pico amperes

Among these IP_3_R-interacting proteins is the anti-apoptotic protein B-cell lymphoma 2 (Bcl-2) [14, 15], involved in inhibiting the mitochondrial apoptosis pathways by preventing Bcl-2-associated x protein (Bax)/Bcl-2 antagonist killer 1 (Bak) activation and thus inhibiting the arising mitochondrial permeabilization and cell death [16, 17]. Structurally, Bcl-2 proteins are comprised of four α-helical domains, also known as Bcl-2 homology (BH) domains (BH1–4, Figure 1A). Of these, the BH1–3 domains are referenced separately as the “hydrophobic cleft”, which binds the BH3 domain of pro-apoptotic Bcl-2 family members and is targeted by BH3-mimetic drugs. This hydrophobic cleft is separated from the BH4 domain by an unstructured loop region (referred to as “Bcl-2 loop”, “loop region” or, simply, “loop” throughout the text, Figure 1A, brown). Additionally, Bcl-2 has a transmembrane domain (TMD) that targets it to intracellular membranes [16]. Multiple studies [18–20] have shown that Bcl-2 is also associated with the ER, where it can interact directly with all three IP_3_R isoforms, inhibiting their activity [21–23]. This interaction plays an important role in the regulation of cell death of many cancer cell lines, such as lymphoma, lung, and leukemia [24, 25]. Disrupting this complex regulation of IP_3_Rs by Bcl-2 and using IP_3_R-derived peptides could overcome apoptosis avoidance, one of the hallmarks of cancer [25, 26], and is, thus, of core interest for the development of novel anti-cancer strategies [27]. Also, Bcl-Xl, another anti-apoptotic Bcl-2-family member, has been implicated in the control of Ca^2+^-driven apoptosis through direct inhibition of IP_3_R channels, a feature that seems to contribute to the cell death resistance of triple-negative breast cancer cells [28].

One of the established models of how Bcl-2 suppresses Ca^2+^ release from the ER via the IP_3_R involves a direct interaction between the BH4 domain of Bcl-2 and at least 2 IP_3_R regions, including a 20 amino acids (aa) region (aa 1389–1408) located in the central modulatory region of IP_3_R [20, 22] and the N-terminal ligand-binding domain of IP_3_Rs (aa 1–604 of IP_3_R1) [29]. However, given the multiple regulatory domains of each protein, it has been suggested that the IP_3_R:Bcl-2 interaction may involve additional interaction sites [30]. In particular, the roles of both the TMD and the hydrophobic cleft of Bcl-2 were investigated. However, only the TMD appeared critical for the efficient Bcl-2-mediated inhibition of IP_3_R-dependent Ca^2+^ release, by binding to the C-terminus of IP_3_R [30]. In line with this observation, the BH3-mimetic drug, ABT-199 or venetoclax, selectively binds the hydrophobic cleft of Bcl-2 with sub-nanomolar affinity [31] and prevents its anti-apoptotic effect on mitochondria, which had no significant effect on intracellular Ca^2+^ release in both Bcl-2-dependent cancer cells and in normal, healthy cells such as pancreatic acinar cells [32, 33]. Furthermore, ABT-199 did not alleviate the inhibition of IP_3_R-mediated Ca^2+^ release brought about by Bcl-2 overexpression [30]. However, these studies only addressed the impact of ABT-199 on global IP_3_R-mediated Ca^2+^ signaling in these cell systems and do not exclude subtle and/or transient changes in IP_3_R activity that the drug might provoke.

In this study, we aimed to investigate how ABT-199 by occupying Bcl-2’s hydrophobic cleft affects the activity of IP_3_Rs near the threshold of channel opening by combining single-channel measurements using the recently developed organelle membrane-derived (OMD) patch-clamp approach [34] with molecular dynamic (MD) simulation studies and circular dichroism (CD). We now report that ABT-199 binding to the hydrophobic cleft of Bcl-2 induces a significant and crucial change in Bcl-2 stability and conformation and in IP_3_R single-channel kinetics. Further, an MD simulation predicts that upon ABT-199 binding, a rearrangement occurs in BH4, at the opposite side of Bcl-2. This conformational change of BH4 is likely responsible for the observed changes in IP_3_R activity: the rearrangement of an N-terminal tail of Bcl-2 induced by ABT-199 binding likely disrupts the ongoing inhibitory interaction of Bcl-2 and IP_3_R, followed by Bcl-2 rebinding with lower inhibitory action.

## Materials and methods

### Cell culture and transfection

Bcl-2-overexpressing WEHI7.2 cells were a kind gift of Prof. C. Distelhorst. Cell culture, transfection, and cloning of Bcl-2 in WEHI7.2 cells were carried out as reported [35].

### Reagents and lipids

ABT-199 (purity > 99.5%) was purchased from Chemietek (Indianapolis, USA). ABT-199 stock solutions were prepared at a final concentration of 10 mmol/L in 100% dimethyl sulfoxide (DMSO) from Sigma-Aldrich (Missouri, USA; case No.: 67-68-5).

IP_3_ was purchased from Avanti^®^ Polar Lipids, Inc. (Alabama, USA). IP_3_ stock solutions were prepared at a final concentration of 10 mmol/L in H_2_O. Cholesterol powder was purchased from Sigma-Aldrich (case No.: 57-88-5). Cholesterol stock solutions were prepared at a final concentration of 100 mmol/L in 100% chloroform. 1,2-diphytanoyl-sn-glycero-3-phosphocholine (DPhPC) was purchased from Avanti^®^ Polar Lipids, Inc. (Alabama, USA). DPhPC was ordered directly in chloroform solution at a concentration of 30 mmol/L.

### Preparation of ligands and proteins

Solution nuclear magnetic resonance (NMR) spectroscopy structure of the human Bcl-2 isoform 1 [protein data bank (PDB) entry 1G5M] has been used as the initial structure most representative of the full-length Bcl-2 among available structures. The initial structure of the ABT-199 has been based on the published crystallographic data of the closest available analog 4-[4-({4’-chloro-3-[2-(dimethylamino)ethoxy]biphenyl- 2-yl}methyl)piperazin-1-yl]-2-(*1H*-indol-5-yloxy)-*N*-({3-nitro-4-[(tetrahydro-*2H*-pyran-4-ylmethyl)amino] phenyl}sulfonyl)benzamide [19], PDB entry 1Y1, by replacing the O_63_ group, linked to C_29_ with two CH_3_ groups linked to the C_9_ atom, to recreate the appropriate ABT-199 chemical structure (Figure S1). In order to study the interaction of the ABT-199 with Bcl-2, the ABT-199 molecule has been positioned in the proximity of the BH3 hydrophobic cleft in 5 different positions (3 in close proximity, within 2–3 Å, and two others with the increasing distance of 5 nm and 1 nm, as illustrated on Figure S2).

### Electrophysiology and solutions

Isolation of the ER-containing membrane fractions from Bcl-2-overexpressing WEHI7.2 cells and preparation of the giant unilamellar vesicles (GUVs) were carried out using OMD patch-clamp technique as described previously [30]. GUVs were prepared from the 1:5 mixtures of the ER-containing fraction with a 10:1 diphytanoylphosphatidylcholine/cholesterol lipid combination (5 mmol/L). The patch-clamp experiments were carried out using Axopatch 200B amplifier and pCLAMP 10.0 software (Molecular Devices, Union City, CA) for data acquisition and analysis. Patch pipettes were fabricated from borosilicate glass capillaries (World Precision Instruments, Inc., Sarasota, FL) on a horizontal puller (Sutter Instrument Company, Novato, CA) and had a resistance in the range of 7–10 mΩ. Prepared vesicles were immersed in a bath solution containing 150 mmol/L cesium chloride (CsCl), 10 mmol/L HEPES, 1 mmol/L MgCl_2_, 2 μmol/L free CaCl_2_ [0.9 mmol/L CaCl_2_ + 1 mmol/L ethylene glycol-bis(β-aminoethyl ether)-N,N,N′,N′-tetraacetic acid (EGTÅ)], pH 7.2. Patch pipettes were filled with the same solution.

### CD experiments

6xHis-Bcl-2 proteins were purified as described in the study [36]. Proteins were dialyzed in 5 mmol/L 3-(*N*-morpholino)propanesulfonic acid (MOPS) pH 7.5, 5 mmol/L NaCl, for 15 h, at 4°C; 3× changes; constant stirring. Aggregated material was removed by centrifugation (20,000 ×g; 15 min; 4°C) before protein concentration was determined on a NanoDrop 2000 instrument (Thermo) using the absorbance at A_280_ in the linear part of the instrument’s dynamic range. The molecular extinction coefficient and weight for the A_280_ analysis were calculated using the Expasy server (http://web.expasy.org/protparam/).

Variable temperature measurements (10°–90°C; 1°C/min) at 222 nm and near-ultraviolet (UV) spectra (320–260 nm) were recorded on a Jasco (Japan) J-1500 spectropolarimeter, equipped with a Peltier temperature control element and a six-position cuvette holder. Samples of 15 μmol/L protein were monitored in 5 mmol/L MOPS (Sigma-Aldrich, Missouri, USA) pH 7.5; 5 mmol/L NaCl; 1mmol/L dithiothreitol (DTT); 0.5% DMSO, without/with the indicated ABT-199 concentrations, in 1 mm quartz cuvettes (Hellma GmbH & Co. KG, Müllheim, Germany); data pitch: 0.5 nm; bandwidth: 1 nm; scanning speed: 50 nm/min; DIT: 0.5 s; accumulation: 3. Molar helipticity was determined using the Jasco software. The apparent melting temperature (*Tm_app_*) was derived by acquiring the first derivatives of the melting curves, using the calculus function of Origin 7.0 software (OriginLab, MA USA).

### Data analysis

Two different groups of programs were used for the analysis of single-channel data: pCLAMP 10.2 (Molecular Devices, CA, USA) and QuB 2.0.0.8 [31, 32]. Origin 7.0 was also used for some of the data fitting and plotting. The analysis and simulation of single-channel recordings were performed as detailed in the following sections.

### Number of channels, conductance, idealization and stability analysis

Traces suitable for single-channel analysis were selected by sorting only recordings showing the activity of one channel. Observing IP_3_-stimulated activity for at least 5 min and taking mean open and closed dwell times to be 7 ms and 637 ms, respectively, the probability that two identical channels would never exhibit multiple conductance levels was < ~10^–37^ [37], which demonstrates the validity of this rejection criterion.

Any baseline drift was manually corrected. The recorded activity was quantified by performing a single-channel search analysis using the Clampfit-10 program (pCLAMP software suit, Molecular Devices) and QuB 2.0 programs as described previously [38, 39]. Single-channel conductance at the various voltages was measured by visually setting cursors at the baseline and open channel current level for computer measurement of those openings of sufficient duration such that filtering effects on amplitude should be minimal [40]. The traces were idealized using two different methods: 50% half amplitude [40] and segmental K means [38].

### MD simulation

The prepared protein structures of the Bcl-2 alone or in complex with ABT-199 have been solvated in the dodecahedral box with margins of 2 nm and periodic boundary conditions, and the total electrical charge of the system has been neutralized by the addition of 10 or 9 Na^+^ ions for Bcl-2 alone or in complex with ABT-199 correspondingly [41]. The prepared systems have been equilibrated by performing steps of energy minimization, followed by reheating the system to 300 K and pressurizing the system at 1 bar under NVT and NPT ensemble MD runs with restricted Bcl-2 and ABT-199 structures [42]. MD simulations were carried out on the prepared systems enclosing Bcl-2 alone or in a complex with ABT-199 for 100 ns. All calculations were carried out using GROMOS96 54A7 force field [43].

## Results

### ABT-199 relieves inhibition of IP_3_R activity by Bcl-2 in a complex manner

We recently investigated the possible interaction of the hydrophobic cleft of Bcl-2 with IP_3_R using ABT-199, which specifically targets this domain (Figure 1A, 1B). When IP_3_R was stimulated above basal levels by 5 μmol/L IP_3_, we found no significant changes in IP_3_R activity upon ABT-199 administration [30]. To complement these studies, we tested the effect of ABT-199 on IP_3_R regulation under conditions mimicking the basal activity of IP_3_R. Single-channel IP_3_R activity was measured using the OMD patch-clamp technique [35] using a WEHI7.2 cell line that expresses IP_3_R and overexpresses Bcl-2 (Figure 1B). First, basal single-channel IP_3_R activity was acquired in the presence of 2 μmol/L (Figure 1C) or 5 μmol/L [IP_3_] (Figure 1D), followed by the addition of 1 μmol/L ABT-199, a concentration sufficient for binding Bcl-2 in several cancer cell lines with a lethal dose 50 (LD_50_) of about 10 nmol/L [33, 44]. No IP_3_R activity could be observed in the absence of IP_3_, and the application of ABT-199 did not evoke any response either (Figure S1A).

In line with our previous results [30], ABT-199 did not have any significant effect on IP_3_R activity triggered by 5 μmol/L IP_3_ (Figure 1D). However, at 2 μmol/L IP_3_ stimulation, the application of ABT-199 altered the IP_3_R activity pattern (Figure 1C). Following the basal, low P_o_ of ~10^–3^ (Figure 1C, “basal” region) activity, application of ABT-199 produced a short (typically under 30 s) burst of IP_3_R activity, resembling that of uninhibited IP_3_R in WEHI7.2 cells lacking Bcl-2 [34]. This was followed by a prolonged period of activity with a modest but significantly higher P_o_ than during the basal period. These observations suggested that ABT-199 induces a small but measurable change to IP_3_R gating properties and raised the possibility that this could be due to alterations of the IP_3_R:Bcl-2 interaction. The specificity of these changes in IP_3_R gating the IP_3_R:Bcl-2 interaction was verified by testing the effects of ABT-199 application to the GUVs prepared from the extracts from the native WEHI7.2 cells, which express only IP_3_R and sub-detection level of Bcl-2 (Figure S1B) [26, 31]. Additionally, following the same procedure as above for ABT-199, we have tested wehi-539 which, at the utilized 5 nmol/L concentration, specifically antagonizes Bcl-Xl (a protein that can also inhibit IP_3_Rs), but not Bcl-2 (Figure 1E). Neither additional test has evoked a complex activity pattern described above.

### ABT-199 decreases the energy difference between open and closed kinetic states of IP_3_R

**Figure 2.**
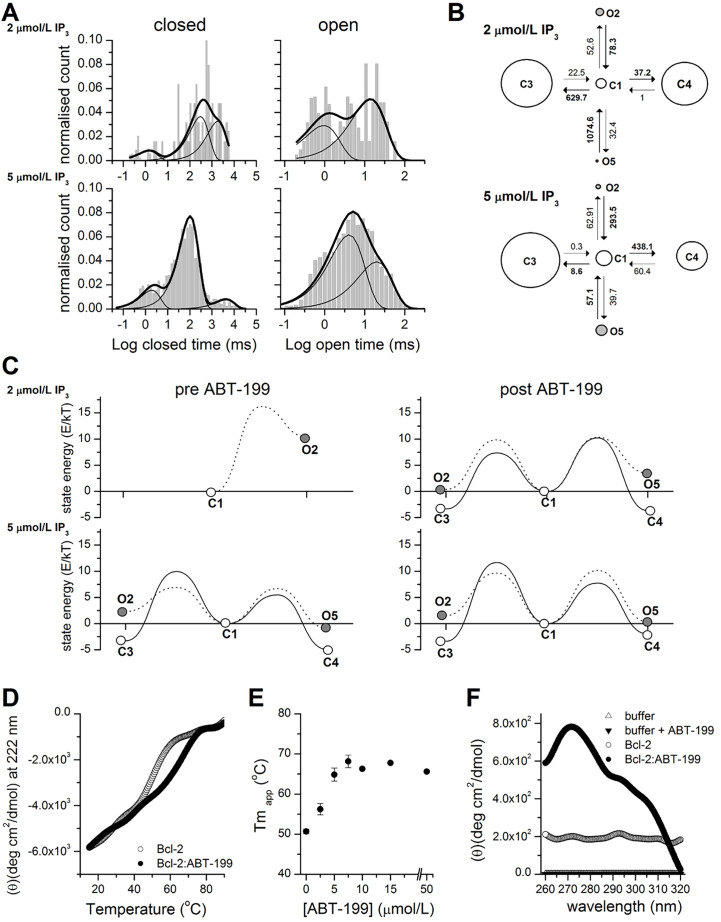
ABT-199 significantly affects the stability of Bcl-2 and the opening energy of Bcl-2 bound IP_3_R kinetic states. (A). Representative distributions of closed (left) and open (right) dwell times in the single-channel traces of IP_3_R activity stimulated by 2 μmol/L or 5 μmol/L IP_3_ as indicated after application of 1 μmol/L ABT-199 (*n* = 4 for both concentrations). The histogram shows the distribution of dwell times. The thick continuous line represents the cumulative best fit corresponding to the kinetic model used and broken lines represent individual components of the fit. (B). Kinetic models providing the best fit to the IP_3_R inhibited by Bcl-2 activity stimulated by 2 μmol/L or 5 μmol/L IP_3_ and following the application of ABT-199. Letters C and O denote closed and open kinetic states. The area of each circle is proportional to the log of total time spent in the corresponding state. Kinetic rates (s^–1^) are indicated as numbers associated with the corresponding arrows indicating interstate transitions. (C). Energy landscape plots summarizing the relative energies of closed and open states calculated from the corresponding kinetic models at indicated [IP_3_] before (left) or after (right) ABT-199 application. Solid lines connecting C1 to open circles represent energy barriers for the transition between closed states and dotted lines represent transitions from C1 to open states. (D). Thermal denaturation curves (15°–90°C) obtained by monitoring ellipticity at 222 nm, by far-UV CD, while heating (at 1°C/min^−1^) the Bcl-2 protein samples (15 μmol/L) in the presence or absence of 15 μmol/L ABT-199 (as indicated). A representative experiment is shown, following smoothing (Origin; FFT filter 15; *n* = 3). (E). The *Tm_app_* of Bcl-2 protein in the absence or presence of the indicated ABT-199 concentration was determined from experiments performed under conditions described in panel D. (F). Near-UV CD spectra recorded for Bcl-2 (15–20 μmol/L) in the absence (empty circles) or presence (filled circles) of 50 μmol/L ABT-199. Control-spectra were recorded for buffer alone (empty triangles) or buffer plus ABT-199 (filled triangles) under identical conditions. A representative experiment, following smoothing, is shown (*n* = 4)

To characterize in detail this pattern of ABT-199-driven IP_3_R activity, we performed a kinetic analysis of IP_3_R current traces before and after the application of ABT-199 (Figure 2). The acquired traces were scrutinized for the appearance of multiple conductance levels. The selected traces of sufficient duration and containing only single-channel activity with sufficient confidence (typically *P* < 10^–10^) [37] were individually analyzed, as described [38, 39]. Analysis of open and closed dwell time distributions revealed a presence of at least 3 closed and 2 open states (Figure 2A, 2B), in good agreement with earlier studies [45]. These studies proposed a kinetic model that described IP_3_R gating properties using a multi-modal description of IP_3_R bursting behavior at different stimulation levels. A subset of such behaviors could be observed by us at fixed concentrations of Ca^2+^ and IP_3_, corresponding to a subset of conditions investigated in the study [45]. Representative dwell time distributions and kinetic models describing IP_3_R gating in the presence of 2 μmol/L or 5 μmol/L Ca^2+^ and following ABT-199 application are shown in Figure 2A and 2B. See also Table S1 for a full set of kinetic parameters describing IP_3_R gating under all investigated conditions. The energy landscapes representing IP_3_R activity before and after the application of 1 μmol/L ÅBT-199 at 2 μmol/L and 5 μmol/L [IP_3_] are summarized in Figure 2C. As can be seen, the application of ABT- 199 led to a decrease in the gap between closed and open state energies (Figure 2C). This was especially evident at 2 μmol/L [IP_3_] (Figure 2C, top) which was due to the low basal P_o_ of 0.0077 ± 0.0063 and the correspondingly small number of total events, and it was impossible to reliably estimate all parameters of a complete 3 closed and 2 open state model. As a result, the corresponding energy landscape is represented by a simplified model consisting of single open and closed states with a significant energy difference of 10.4 Kt ± 0.2 Kt (or 42.8 pN/nm ± 1 pN/nm at room temperature). Åt 5 μmol/L [IP_3_] (Figure 2C, bottom), however, this effect was masked by a significantly higher overall P_o_ (0.26 ± 0.09) and, correspondingly, a lower difference in energies between the closed and open states (Figure 2C).

These observations prompted us to consider the possibility that the addition of ABT-199 and its binding to Bcl-2 impacts the structural arrangement of the Bcl-2 protein, particularly in the BH4 domain.

### ABT-199-driven stabilization of Bcl-2

To investigate how the binding of ABT-199 to its hydrophobic cleft may affect the stability of Bcl-2, we analyzed purified 6xHis-Bcl-2 in the presence or absence of ABT-199, by CD spectral analysis in the far-UV at increasing temperatures (Figure 2D). 6xHis-Bcl-2 displays the characteristic spectrum of an α-helical protein with minima at 208 nm and 221 nm [46]. Collecting data at 222 nm during thermal ramping allows the determination of *Tm_app_*, which serves as a direct indicator of protein stability (Figure 2D). 6xHis-Bcl-2 is very stable with a *Tm_app_* of 50.6°C ± 0.5°C in the absence (not shown) or presence of 0.5 *v*/*v*% DMSO solvent (Figure 2D, grey) [37]. Addition of ABT-199 (0.5 *v*/*v*% DMSO final concentration) shifted the *Tm_app_* to 67.78°C ± 0.18°C) (Figure 2D, black). The *Tm_app_* stabilization was dependent on ABT-199 concentration and reached a plateau at a 1:1 molar ratio (Figure 2E). These data indicate that ABT-199 significantly stabilizes the structure of Bcl-2 overall. Analysis of near-UV spectra can provide information on the 3-dimensional (3D) structure of a polypeptide and is sensitive to even small changes in the structure, by monitoring the environment of Trp, Phe, and Tyr residues [47, 48]. Clearly, the near-UV spectrum of Bcl-2 shifted upon ABT-199 addition but not by the addition of buffer alone (Figure 2F). Therefore, ABT-199 potentially impacts the local environment of one or multiple aromatic aa probes of the protein. This would be consistent with a measurable, ABT-199-driven, tertiary conformational change in the protein.

### MD simulation of the Bcl-2:ABT-199 interaction reveals a difference in most commonly observed Bcl-2 configurations

To gain structural insight on how Bcl-2 may be affected by ABT-199 binding, we performed MD simulations of the Bcl-2 protein alone and in complex with ABT-199. Among the available structures, the NMR spectroscopy structure of the human Bcl-2 (PDB entry 1G5M) was used as the initial structure representative of Bcl-2. This structure has a partially truncated loop (missing residues 51–91), while in all other available structures of Bcl-2 it is completely absent [49–51]. In view of the presence of a partial loop and the presence of a complete hydrophobic cleft, this structure was judged to be the most appropriate for further study.

**Figure 3.**
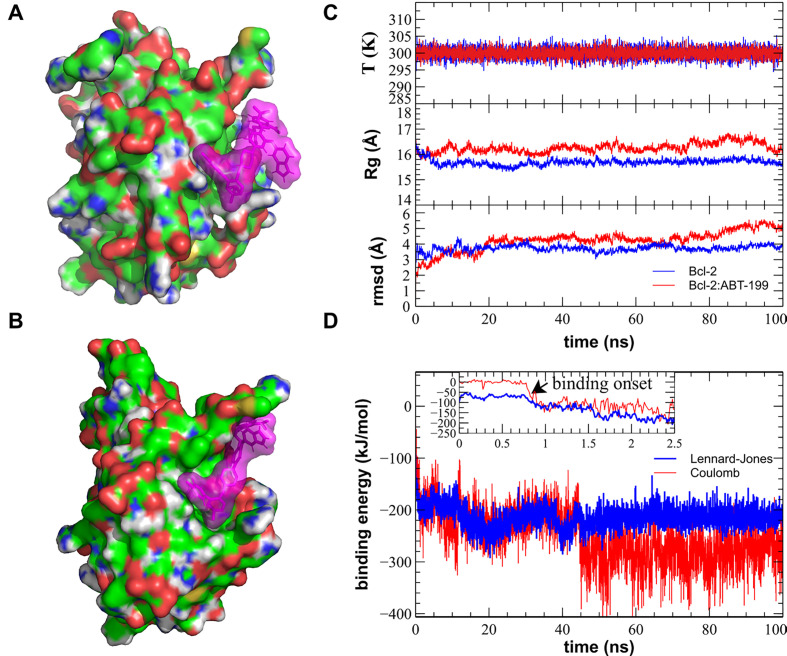
MD simulation of the Bcl-2:ABT-199 interaction produces a successful binding of the ABT-199 to the hydrophobic cleft of the Bcl-2. (A). Sample initial position of the ABT-199 (violet semitransparent spheres) in the proximity of the crystal structure-based Bcl-2 (CPK-colored surface). (B). A representative simulation frame showing the Bcl-2:ABT-199 complex following the binding event and equilibration. (C). Representative curves showing stable evolution of the system temperature (top), Bcl-2 gyration radius (Rg, middle), and root mean square deviation (rmsd, bottom) during the entire duration of the MD simulation. (D). Time dependence of the binding energies (Coulomb force in red and Lennard-Jones potential in blue) during the course of the MD simulation. Note the start of the binding event commencing within the first ns of the simulation, typical of the ABT-199 prepositioned in the close proximity of the BH3 hydrophobic cleft, while the propagation of conformational change induced by this binding and equilibration of Bcl-2 structure continuing for additional ~40 ns. T: temperature

The structure of ABT-199 used for modeling was based on its closest analogue for which crystallographic data are available (PDB entry 1Y1) [19], adapted by replacing the O_63_ group, linked to C_29_ with two CH_3_ groups linked to the C_9_ atom (Figure S2). To study the interaction of ABT-199 with Bcl-2, the former was positioned in the proximity of the hydrophobic cleft of Bcl-2 in five different positions (three in close proximity, within 2–3 Å, and two others with an increasing distance of 0.5 nm and 1 nm, one example shown in Figures 3A, S3). Next, the structures of free Bcl-2 or the Bcl-2:ABT-199 complex were solvated, pressurized, and prepared as described [41, 42]. MD simulations (four for Bcl-2 alone, and five for Bcl-2:ABT-199 complexes, all with independent randomized solvation and ionic neutralization), were carried out on these systems for 100 ns (Figures 3, S2). All calculations were carried out using the GROMOS96 54a7 force field [43].

Representative MD simulation runs show initial and final configurations following the binding event (Figure 3A, 3B). All controls of the major stability parameters, such as system temperature, Bcl-2 Rg, and rmsd from the starting structure, remained stable throughout the runs (Figure 3C). Visual observation confirmed that during the entirety of the performed MD runs, the simulated systems remained stable with Bcl-2 retaining its secondary and tertiary structures (Supplementary material). Binding of ABT-199 to Bcl-2 commenced within the 1st ns for all three initial configurations in which ABT-199 had been placed in close proximity of hydrophobic cleft (Figure 3C), as revealed by Bcl-2:ABT-199 energy interaction graphs (Figure 3D, Movie S1). The other configurations, in which ABT-199 was placed further away from the hydrophobic cleft, took significantly more time to arrive at close contact between the interacting molecules. However, they still lacked signs of stabilization of interaction energy by the end of the run (data not shown) and were, thus, discarded.

**Figure 4.**
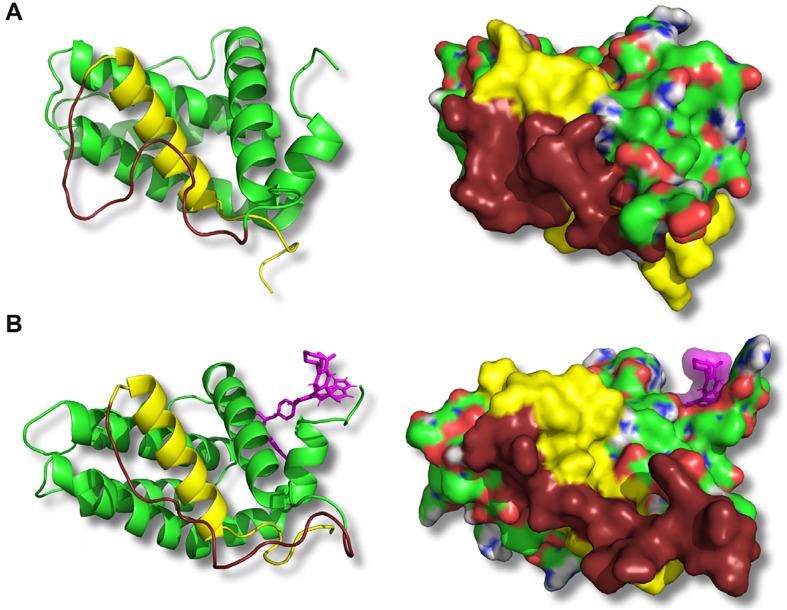
ABT-199 binding to the BH3 hydrophobic cleft leads to conformational changes on the opposite side of Bcl-2 in the BH4 domain. Representative structures corresponding to the medians of principal structure clusters of the free Bcl-2 (A) or Bcl-2:ABT-199 complex (B) following the ABT-199 binding (in B) and equilibration are shown. Panels show a ribbon representation of the backbone (left) or the CPK-colored surface (right) of the Bcl-2 protein. The BH4 domain is highlighted in yellow, and the adjacent loop is in dark brown. ABT-199 is represented with violet sticks (left) or a semitransparent surface (right). Note a difference in BH4 domain structure (both backbone and exposed surface) as well as adhesion of a part of the loop between BH4 and BH2 domains in the Bcl-2:ABT-199 complex

The representative configurations of Bcl-2 alone or in a complex with ABT-199 were extracted from the trajectories following the binding event and binding energy equilibration (post 50 ns universally, Figure 3D) by performing a clustering analysis with a cut-off rmsd of 1.5 Å on the collected sets of structures [52]. For each run, the median structure of the maximal size cluster has been selected to represent the most common structure of the Bcl-2 or Bcl-2:ABT-199 complex. The median structures of the extracted clusters for different MD runs were consistently reproducible between structures in matching conditions (Bcl-2 alone or Bcl-2:ÅBT-199) with rmsd of no more than 2.5 Å within matching groups of clusters (representative median structures of free Bcl-2 and Bcl-2:ABT-199 are compared in Figure 4). The dynamic structure of free Bcl-2 remained stable, preserving not only the relative positioning of all four BH domains but also the separation of the loop from the rest of Bcl-2. It should be noted that the loop exhibited a high degree of variability throughout the runs, as expected for a region lacking a rigid secondary structure. Nonetheless, in the case of the Bcl-2:ÅBT-199 complex, the loop was partially stabilized, rendering 10 of its aa proximal to the α-helix of the BH4 domain. This rearrangement partially obscures the moiety of BH4 that is likely to participate in the interaction of Bcl-2 with IP_3_R [20].

#### ABT-199 binding induces a tail-flip change in BH4 and rearranges the Bcl-2 loop, partially obscuring the BH4:IP_3_R interaction

**Figure 5.**
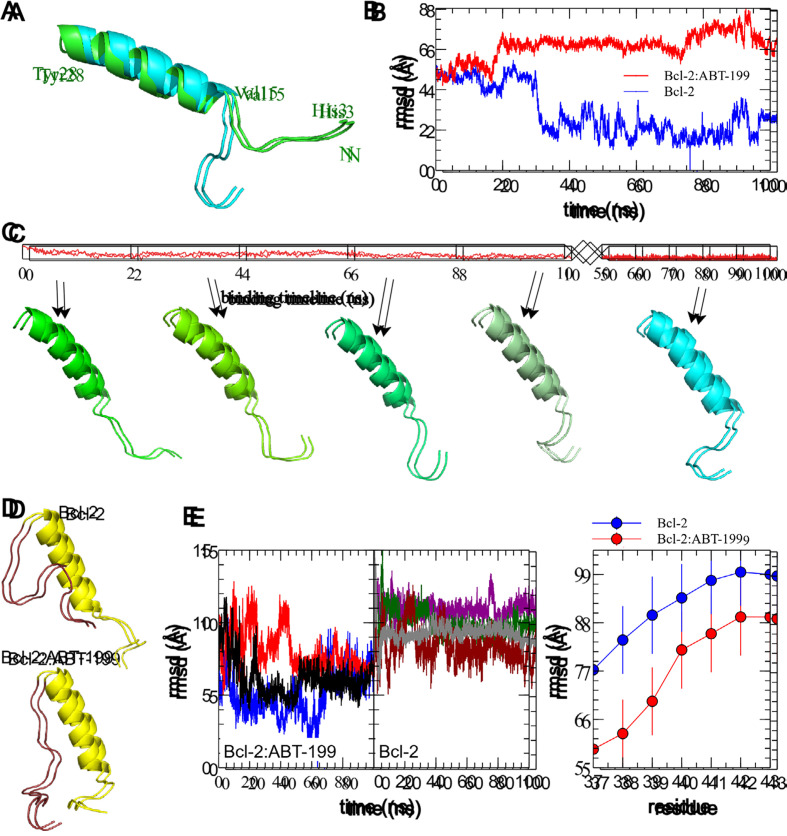
ABT-199 binding to Bcl-2 induces an N-terminal tail-flip event and rearranges the Bcl-2 loop. (A). Comparison of the median structures of the principal clusters of BH4 domain structures in free Bcl-2 post equilibration (green backbone) and ABT-199 bound Bcl-2 following the binding event (cyan backbone). N-terminus of Bcl-2 is denoted with the green letter N, and endpoints and α-helix of the represented fragment are marked in green with corresponding residue labels. Note the stabilized “tail-flipped” configuration of the BH4 domain in the ABT-199 bound structure. (B). rmsd variation in the BH4 domain of the Bcl-2 alone and interacting with ABT-199. Note the small deviation from the most common structure of the free Bcl-2 after a short equilibration period, compared to a significantly larger mean rmsd in ABT-199 bound Bcl-2 following a noticeably longer period of propagation of the conformational change post the onset of the binding. (C). Representative frames illustrating the propagation of the conformational change following the onset of ABT-199 binding. (D). Comparison of Bcl-2 fragment median structures, including BH4 and loop domains in Bcl-2 alone (top) and Bcl-2:ABT-199 complex (bottom). Note the realignment of the loop fragment immediately adjacent to BH4. (E). Individual plots of rmsd of the first 10 residues of the Bcl-2 loop (residues 29–39) with regard to median structures of the Bcl:ABT complex (left) and, evolution of average rmsd values as a function of loop fragment length, indexed by the last residue (right). Note significantly smaller deviations of the Bcl:ABT complex loop fragments up to residue 39, representative of the loop region adhering to the BH4 α-helix

To investigate further the apparent rearrangement of the Bcl-2 structure in the proximity of BH4, we analyzed how the structure of free Bcl-2 or the Bcl-2:ABT-199 complex changes in the BH4 vicinity following ABT-199 binding (50–100 ns trajectory intervals for both trajectory types). Median structures of the major clusters derived as above were compared for each condition (Figure 5A, 5B). In these, the α-helical part of BH4 (residues 15–30) shows only a limited residue displacement and reorientation. The most striking difference can be observed in the N-terminal region of Bcl-2 following ABT-199 binding and can be best described as a turning of the tail formed by the N-terminal residues of Bcl-2 that precede the BH4 α-helix (aa 1–14, Figure 1A). This is also illustrated by the sequence of frames of representative BH4 structures at different times during this evolution initiated by ABT-199 binding (Figure 5C). In contrast, in the free Bcl-2 trajectories, the structure of this tail was significantly less stable, similar to that of the loop.

To determine how this affects the Bcl-2 loop, we performed a series of rmsd comparisons of loop fragments of increasing length for Bcl-2 alone and Bcl-2:ABT-199. The N-terminal domains containing the BH4, and the Bcl-2 loop (residues 1–45) were aligned along the BH4 α-helix (residues 10–28), which was conserved in all runs (rmsd variation within 2 Å). Then the rmsds of the loop fragments of varying lengths (residues 29–X, X = 36–43) were calculated for each run trajectory against the median structure of Bcl-2:ABT-199. These rmsds and the median structures were compared emphasizing the Bcl-2 loop, for the 4 “control” runs of Bcl-2 alone and three Bcl-2:ABT-199 runs (Figure 5D, 5E). The major visible difference between the median cluster structures of the loop fragments (Figure 5D, brown) is that in the case of Bcl-2:ABT-199, the loop is stretched with its first ~10 residues following along the BH4 helix, while in free Bcl-2, the loop exhibits a significantly higher motional freedom. Quantitatively, this can be seen as a significantly smaller rmsd variation for the Bcl-2:ABT-199 loop fragments ending in residues ≤ 39 (Figure 5E, right). This is further supported by a similar analysis of the energies of the interaction of the loop fragments of varying lengths with the BH4 helix (Figure S4).

## Discussion

The BH4 domain of Bcl-2 binds to IP_3_R, inhibiting its activity [20]. This action is facilitated by the TMD domain of Bcl-2 via concentrating Bcl-2 in the proximity of IP_3_R in the ER membrane [30]. On the other hand, the role of the hydrophobic cleft has been primarily associated with the mitochondrial apoptotic pathways [53]. Moreover, the hydrophobic cleft and the BH4 domain are positioned on the opposite sides of the Bcl-2 (Figure 4B). For this reason, it is hard to expect a direct effect of ABT-199 or any of its analogs, known to bind specifically to the hydrophobic cleft, upon Bcl-2/IP_3_R interaction. In line with that, previous studies did not reveal any major changes in the ability of Bcl-2 to suppress IP_3_Rs in response to ABT-199 binding [30, 33, 34]. However, these studies solely studied global Ca^2+^ signaling events in response to agonists that provoke an intermediate level of IP_3_R activity. In contrast, by studying IP_3_R gating at low P_o_ levels, under conditions reminiscent of IP_3_R activity in non-stimulated cells, we detected an unusual pattern of IP_3_R activity upon application of ABT-199. The data suggest that occlusion of the hydrophobic cleft of Bcl-2 by ABT-199 may transiently affect the association of Bcl-2 with IP_3_R. Specifically, the application of ABT-199 provoked a short period of unrestrained (by Bcl-2) IP_3_R activity with P_o_ roughly matching that of IP_3_R under similar conditions but in the absence of Bcl-2 [34, 54], followed by equilibrium “partially inhibited” IP_3_R activity. Kinetic analysis has shown that IP_3_R activity both before and after the ABT-199 application could be described by the kinetic models having the same topology. The main effect of the application of ABT-199 has been to shift energies of closed kinetic states (Figure 2C), decreasing the gap between open and closed states and thus hinting at the possibility that the binding of ABT-199 to Bcl-2 affects its interaction energy with IP_3_R.

To further investigate the role of the hydrophobic cleft in the IP_3_R:Bcl-2 interaction, we complemented these experiments with MD simulations. Simulations of Bcl-2 alone in an aqueous environment show that Bcl-2 preserves its overall structure in the form largely corresponding to that reported in the recently published crystal structure [55]. Namely, the Bcl-2 loop remains mostly open, exposing a significant portion of BH4, thus allowing a tight binding of Bcl-2 with IP_3_R (Figure 4A). It should be noted that our observation of the overall flexibility of the loop is in good agreement with prior crystallographic data which either completely excluded the Bcl-2 loop or included only a significantly shortened partial structure [49–51]. Taken together, these observations suggest the good accessibility of the BH4 contact surface that was experimentally shown to reliably bind IP_3_R in prior studies [20]. In contrast, the binding of ABT-199 to the hydrophobic cleft of Bcl-2 induced a rapid conformational change to the BH4 domain and its vicinity. First, we observed that the binding induced a “tail-flip” change in the N-terminal part of the Bcl-2 (residues 1–15, Figures 4B, 5) in the immediate proximity of BH4 helix and, thus, IP_3_R channel in the case of their interaction. We hypothesize that this ABT-199-driven conformational change may promote the detachment of Bcl-2 from its initially bound state on the IP_3_R. This would relieve the inhibition of IP_3_R activity, leading to the observed transitory “peak zone” in the single-channel current traces (Figure 1C). Another important observation is a rearrangement of the Bcl-2 loop following ABT-199 binding. The loop residues immediately adjacent to BH4 follow along the BH4 α-helix in the Bcl-2:ÅBT-199 complex, unlike in free Bcl-2 (Figure 5D). Note that, while the simulated Bcl-2 structure included only a partial loop region (even in the most complete structure available to date, due to the general instability of the loop domain), this BH4-loop interaction includes only a fraction of the entire loop (~10 residues immediately adjacent to the BH4 domain, out of the 25 available in the structure), with the rest of the loop demonstrating a lack of a stable configuration similarly to Bcl-2 alone.

**Figure 6.**
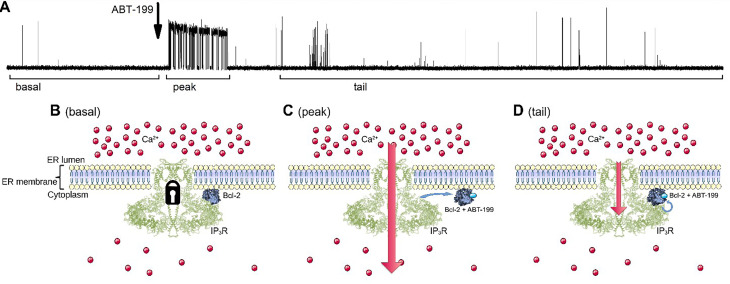
Schematic diagram illustrating the proposed model of how ABT-199 binding to the hydrophobic cleft of Bcl-2 influences IP_3_R activity pattern in panel A. (B). The IP_3_R is inhibited by Bcl-2 via its BH4 and TM domains, yielding a typical low-P_o_ IP_3_R activity at basal stimulation levels. (C). The addition of ABT-199 leads to its binding in the hydrophobic cleft of Bcl-2, which leads to the “tail-flip” conformational change in the BH4 domain which, in turn, disrupts the ongoing inhibitory interaction of Bcl-2 and IP_3_R exposing uninhibited IP_3_R activity for a short period of time. (D). Bcl-2:ABT-199 complex rebinds the IP_3_R, however with lower affinity, yielding the partially inhibited gating pattern. TM: transmembrane

Our observations allow us to propose that ABT-199 binding to the hydrophobic cleft of Bcl-2 affects the BH4 domain located on the opposite side, provoking an N-terminal “tail-flip” event, which may be responsible for disturbing the initial binding of Bcl-2 and IP_3_R. This is followed by a rearrangement of the loop, leading to “rigidification” of the Bcl-2 structure, observed as a stabilization of the Bcl-2 protein in temperature-melting CD measurements (Figure 2D, 2E), and partial obscuring of the BH4 domain that is directly involved in the interaction with IP_3_R. This ABT-199-driven change of the exposed BH4 could be significant enough to terminate an established binding of Bcl-2 to IP_3_R, thus temporarily alleviating IP_3_R inhibition. However, the change is not drastic enough to completely prevent further binding of the ABT-199:Bcl-2 complex to IP_3_R. The rebinding of this complex happens with lower affinity, thus reestablishing the inhibitory effect upon IP_3_R activity, although to a lesser extent. This sequence of events (Figure 6) can provide a likely explanation of the observed transient lack of inhibition of IP_3_R activity after ABT-199 application, as well as the subsequent less potent renewal of IP_3_R inhibition by Bcl-2.

It is important to note that this subtle effect is evident only under certain conditions when Bcl-2 binding sufficiently suppresses IP_3_R activity to overcome its normal stimulation by other factors, such as IP_3_, Ca^2+^, ATP, etc. [54, 56]. For example, the elevation of [IP_3_] from 2 μmol/L to 5 μmol/L increased average P_o_ beyond that threshold, and thus completely obscured the effect of ABT-199 action. In other words, the impact of ABT-199 on the structure of Bcl-2 and subsequently on its ability to inhibit IP_3_R activity may be particularly relevant in non-stimulated cells. Thus, virtually all cells display a constitutive level of IP_3_R-mediated Ca^2+^ signaling in cells that sustain mitochondrial Ca^2+^ transfers and mitochondrial bio-energetics [57]. Furthermore, Bcl-2 has been reported to increase basal IP_3_R-mediated Ca^2+^ oscillations [58]. Such relatively small signals, relevant during basal IP_3_R activity in non-stimulated cells, may be affected by BH3-mimetic drugs analogous to ABT-199.

In conclusion, our data show that ABT-199 can modulate the inhibitory impact of Bcl-2 proteins on IP_3_R channels by impacting the overall Bcl-2 structure, likely resorting effects at the level of the BH4 domain.

## Abbreviations

[IP_3_]: inositol 1,4,5-trisphosphate concentration
aa: amino acids
Bcl-2: B-cell lymphoma 2
BH4: B-cell lymphoma 2 homology 4
Ca^2+^: calcium
CD: circular dichroism
DMSO: dimethyl sulfoxide
ER: endoplasmic reticulum
GUVs: giant unilamellar vesicles
IP_3_: inositol 1,4,5-trisphosphate
IP_3_R: inositol 1,4,5-trisphosphate receptor
MD: molecular dynamic
OMD: organelle membrane-derived
PDB: protein data bank
P_o_: open probability
rmsd: root mean square deviation
*Tm_app_*: apparent melting temperature
TMDs: transmembrane domains
UV: ultraviolet

## Supplementary materials

The supplementary Figures and Tables for this article are available at: https://www.explorationpub.com/uploads/Article/file/100288_sup_1.pdf. The supplementary movies for this article are available at: https://www.explorationpub.com/uploads/Article/file/100288_sup_2.mp4, https://www.explorationpub.com/uploads/Article/file/100288_sup_3.mp4 and https://www.explorationpub.com/uploads/Article/file/100288_sup_4.mp4.

## Declarations

### Copyright

© The Author(s) 2022.
