# Supplementary material for: Allosteric cross-talk between the hydrophobic cleft and the BH4 domain of Bcl-2 in control of inositol 1,4,5-trisphosphate receptor activity
Source: Explor Target Antitumor Ther. 2022 Jun 28;3(3):375–91. doi: 10.37349/etat.2022.00088 (PMC9400710; doi:10.37349/etat.2022.00088)
Supplement: Supplementary file 1 [file etat-03-100288-s001.pdf]

Supplementary Information for

Allosteric cross-talk between the hydrophobic cleft and the BH4 domain of Bcl-2 in control of IP3R activity.

Abigaël Ritaine[1,2,3,*], George Shapovalov[1,2,*$], Nadege Charlene Essonghe[1,2,], Ian de Ridder[3], Hristina Ivanova[3], Spyridoula Karamanou[4], Anastassios Economou[4], Geert Bultynck[3], Roman Skryma[1,2], Natalia Prevarskaya[1,2,$]

[1] Univ. Lille, Inserm, U1003 - PHYCEL - Physiologie Cellulaire, F-59000 Lille, France
[2] Laboratory of Excellence, Ion Channels Science and Therapeutics, Villeneuve d'Ascq, France
[3] KU Leuven, Laboratory of Molecular and Cellular Signaling, Department of Cellular and Molecular Medicine, Campus Gasthuisberg O/N-I bus 802, Herestraat 49, B-3000 Leuven, Belgium
[4] KU Leuven, Department of Microbiology and Immunology, Rega Institute of Medical Research, Laboratory of Molecular Bacteriology, Herestraat 49, B-3000 Leuven, Belgium
[*] Shared first authorship.
[$] Shared last authorship.

This PDF file includes:

Figs. S1 to S4
Table S1
Captions for movies S1 to S3

Other supplementary materials for this manuscript include the following:

Movies S1 to S3

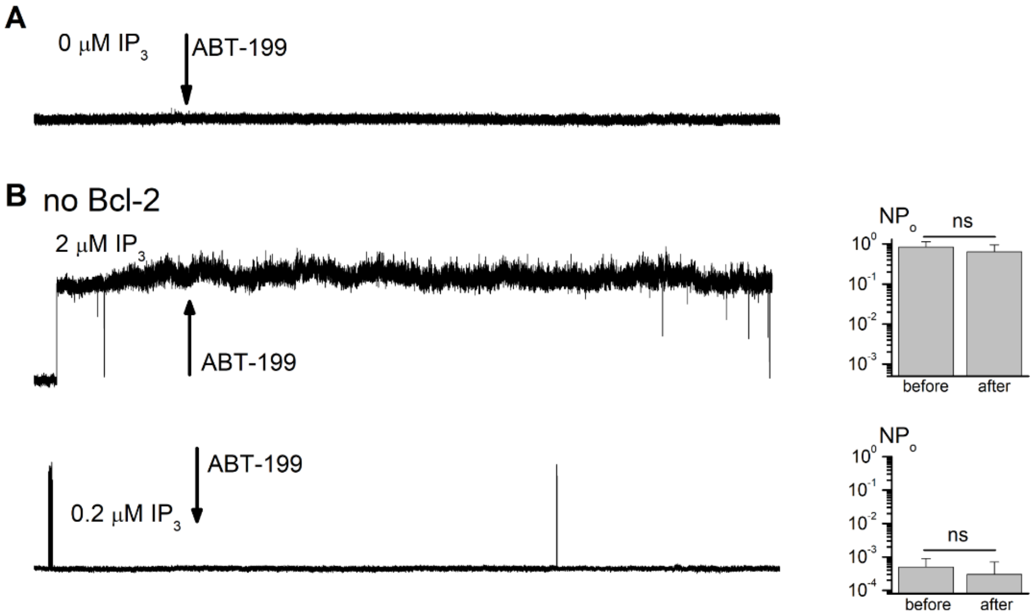

Fig. S1. Controls of the specificity of the ABT-199 effect upon IP3R:Bcl-2 interaction. (*A*) Sample traces showing the effect of application of 1 μM ABT-199 in the absence of IP3. Note complete absence of the IP3R activity before or after the application of ABT-199. (*B*) Sample traces showing the effect of application of 1 μM ABT-199 to the patches exhibiting IP3R activity stimulated by 2 μM IP3 (top) or 0.2 μM IP3 (bottom) in the absence of Bcl-2. Note the absence of ABT-199 effect under either conditions. Barplots on the right of summarize average P_o for the presented conditions (n=5 and 4 correspondingly); ns indicates no significant difference.

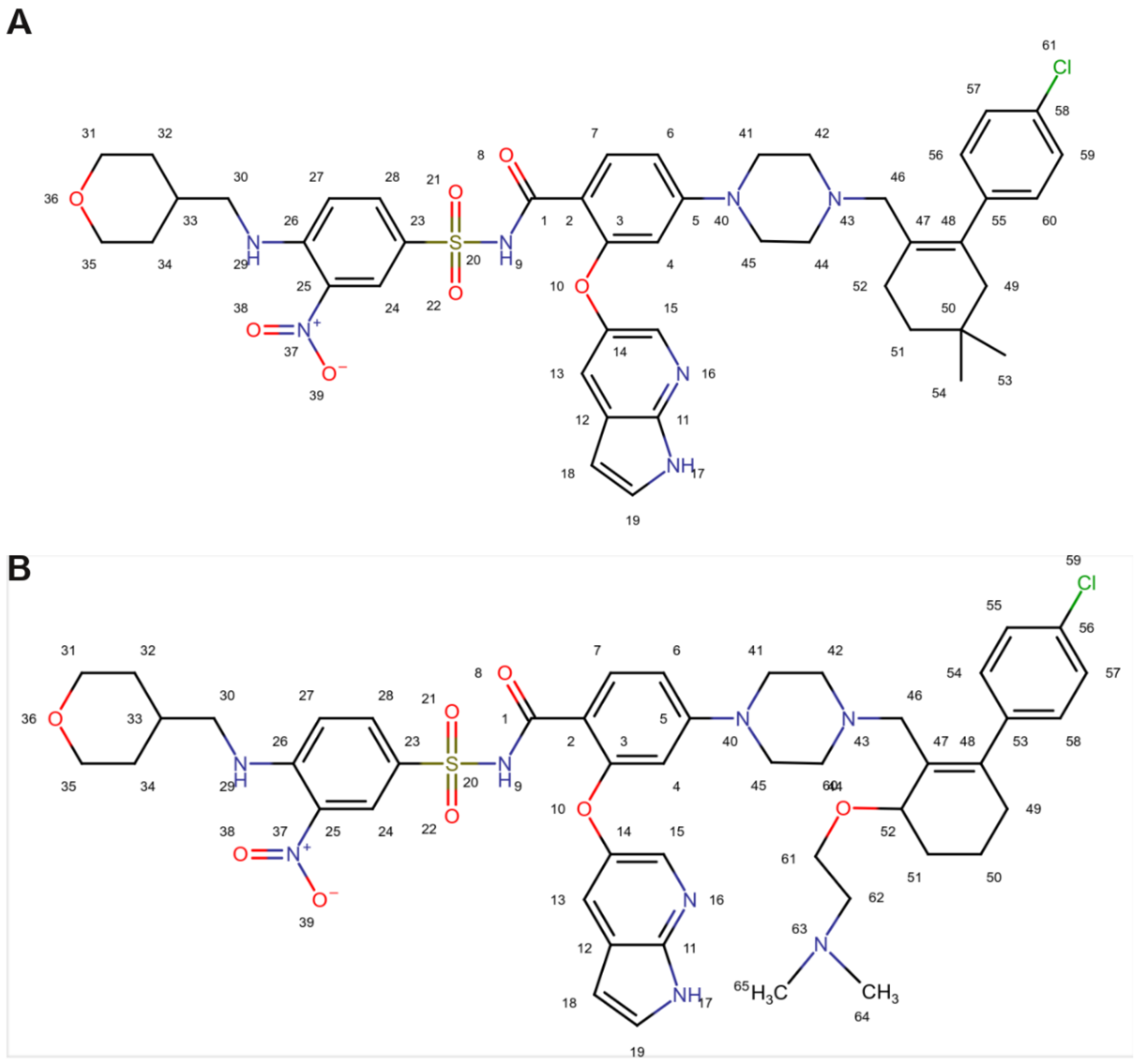

Fig. S2. Comparison of chemical structures of ABT-199 (*A*) with its closest analog with known structure (*B*) (Protein Data Bank entry [1Y1]). In order to obtain a representative 3D structure of the ABT-199 for further MD simulation, a closest analog with published 3D structure has been found and modified in PyMol by removing an unmatched sidechain at C_{52} and adding two carbons at C_{50}, as can be seen on the presented schemes. Schemes were prepared with the help of NIH service [ChemIDplus].

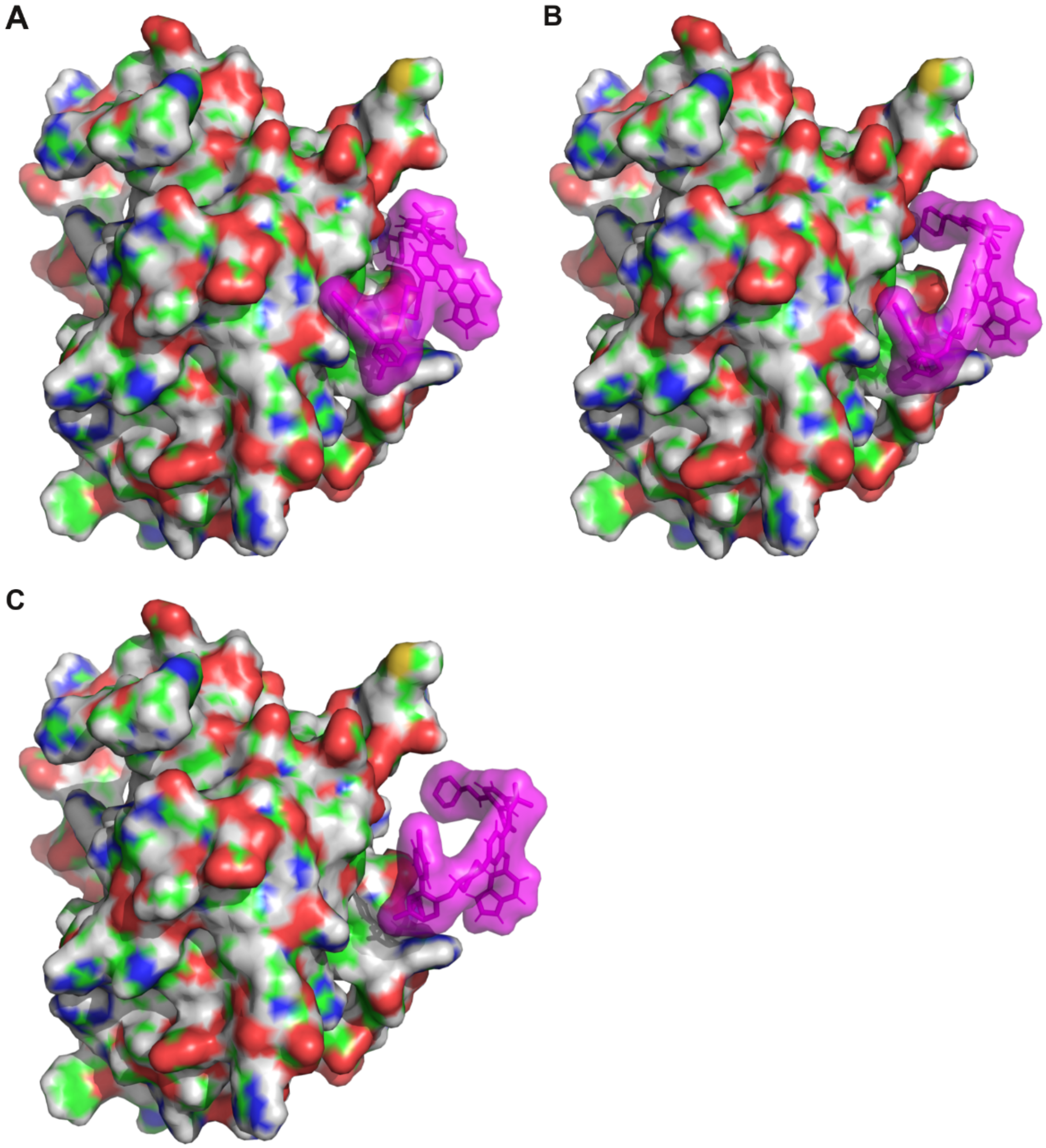

Fig. S3. Illustration of initial positioning of ABT-199 in the proximity of BH3 hydrophobic cleft of Bcl-2. ABT-199 was placed in close (*A*), near (*B*) and far (*C)* proximity (~ 2, 5 and 10 Å respecitvely).

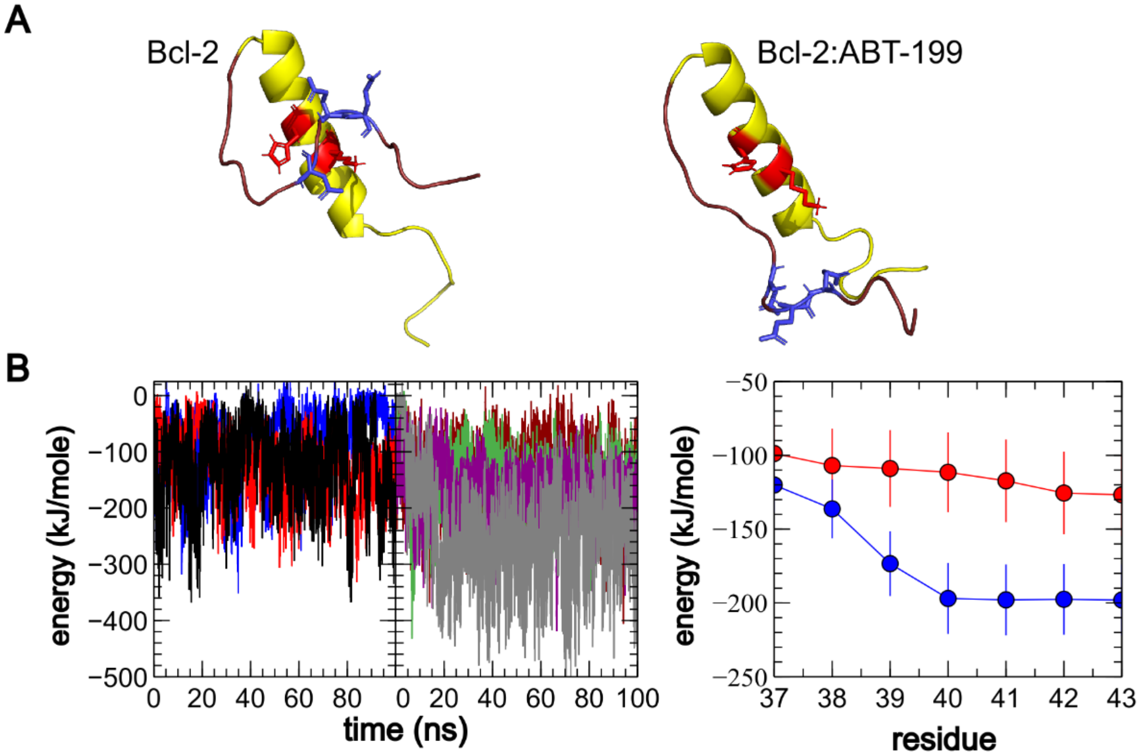

Fig. S4. ABT-199 binding to Bcl-2 disrupts coulomb interaction between charged residues in the loop region and BH4 helix. (*A*) Comparison of Bcl-2 fragment median structures, including BH4 and loop domains, in Bcl-2 alone (top) and Bcl:ABT complex (bottom). Note the realignment of the loop fragment immediately adjacent to BH4 and the disruption of the "hot-spot" interaction of charged residues (interacting residues are shown as sticks highlighted in red for positive His20 and Lys22, and blue for negative Asp35, Glu37 and Glu38). (*B*) (left) individual plots of Coulomb interaction energy between the first 10 residues of the Bcl-2 loop and the BH4 α-helix and, (right) evolution of average Coulomb interaction energies as a function of the loop fragment length indexed by the last residue. Note a significantly smaller Coulomb interaction energy between BH4 helix and loop fragments ending after residue 38 in Bcl:ABT complex, indicating disruption of the "hot-spot" interaction highlighted in panel A.

Table S1. Table representing the different rates and energies for the kinetic models.

Rates (sec^{-1})	2µM IP3 no ABT-199	2µM IP3 + 1µM ABT-199	5µM IP3 no ABT-199	5µM IP3 + 1µM ABT-199
C1→C3		629.73	47.82	8.58
C3→C1		22.45	1.79	0.29
C1→C4		37.19	4226.17	438.11
C4→C1		0.95	29.74	60.35
C1→O2	0.01	52.58	1018.19	62.91
O2→C1	295	78.25	10613	293.49
C1→O5		32.42	1273.9	39.71
O5→C1		1074.64	524.69	57.06

Energy (units of kT)	2µM IP3 no ABT-199	2µM IP3 + 1µM ABT-199	5µM IP3 no ABT-199	5µM IP3 + 1µM ABT-199
C1	0	0	0	0
O2	10.39	0.4	2.34	1.54
C3		-3.33	-3.28	-3.37
C4		-3.67	-4.96	-1.98
O5		3.5	-0.89	0.36

Movies S1 and S2. An illustration of the interaction of Bcl-2 with ABT-199, showing the onset of the binding. Bcl-2 is shown as a CPK colored surface and ABT-199 is represented by semi-transparent violet spheres. Movies show different projections of the first 10 ns of the same trajectory.

Movie S3. An illustration of the changes in the BH4 domain of the Bcl-2 protein, following the ABT-199 binding. The backbone of Bcl-2 protein is shown, with the BH4 domain highlighted in yellow and the first 10 residues of the loop domain highlighted in brown. ABT-199 is shows as violet semi-transparent spheres. Note the tail-flip event in the BH4 domain and the following adhesion of the fragment of the loop region to the BH4 α-helix.
